# Concentration-Dependent Domain Evolution in Reaction–Diffusion Systems

**DOI:** 10.1007/s11538-022-01115-2

**Published:** 2023-01-13

**Authors:** Andrew L. Krause, Eamonn A. Gaffney, Benjamin J. Walker

**Affiliations:** 1 Mathematical Sciences Department, Durham University, Upper Mountjoy Campus, Stockton Rd, Durham, DH1 3LE UK; 2 Wolfson Centre for Mathematical Biology, Mathematical Institute, University of Oxford, Andrew Wiles Building, Radcliffe Observatory Quarter, Woodstock Road, Oxford, OX2 6GG UK; 3 Department of Mathematics, University College London, London, WC1H 0AY UK

**Keywords:** Pattern formation, Evolving domains, Linear instability analysis

## Abstract

Pattern formation has been extensively studied in the context of evolving (time-dependent) domains in recent years, with domain growth implicated in ameliorating problems of pattern robustness and selection, in addition to more realistic modelling in developmental biology. Most work to date has considered prescribed domains evolving as given functions of time, but not the scenario of concentration-dependent dynamics, which is also highly relevant in a developmental setting. Here, we study such concentration-dependent domain evolution for reaction–diffusion systems to elucidate fundamental aspects of these more complex models. We pose a general form of one-dimensional domain evolution and extend this to *N*-dimensional manifolds under mild constitutive assumptions in lieu of developing a full tissue-mechanical model. In the 1D case, we are able to extend linear stability analysis around homogeneous equilibria, though this is of limited utility in understanding complex pattern dynamics in fast growth regimes. We numerically demonstrate a variety of dynamical behaviours in 1D and 2D planar geometries, giving rise to several new phenomena, especially near regimes of critical bifurcation boundaries such as peak-splitting instabilities. For sufficiently fast growth and contraction, concentration-dependence can have an enormous impact on the nonlinear dynamics of the system both qualitatively and quantitatively. We highlight crucial differences between 1D evolution and higher-dimensional models, explaining obstructions for linear analysis and underscoring the importance of careful constitutive choices in defining domain evolution in higher dimensions. We raise important questions in the modelling and analysis of biological systems, in addition to numerous mathematical questions that appear tractable in the one-dimensional setting, but are vastly more difficult for higher-dimensional models.

## Introduction

In proposing his chemical theory of morphogenesis, Turing was clear about the simplifications made to idealize this theory of pattern formation to its core mechanism of a diffusion-driven instability (Turing 1952). In addition to the enormous experimental and theoretical literature exploring this mechanism, an important avenue of research has been extending Turing’s simple theory to ever-more-realistic scenarios incorporating extensions of reaction–diffusion models. For example, there is now a wide literature studying pattern formation in stochastic Turing systems (Woolley et al. 2011; Erban and Jonathan Chapman 2019; Adamer et al. 2020), mechanical and mechano-chemically coupled models (Murray and Oster 1984a, b; Oster et al. 1985; Murray 2004; Vaughan Jr et al. 2013), gene-expression time delays in reaction–diffusion systems (Gaffney and Monk 2006; Seirin Lee et al. 2010; Sargood et al. 2022), cross-diffusion and other generalized transport mechanisms (Ritchie et al. 2022), reaction–diffusion patterning on manifolds and networks (Plaza et al. 2004; McCullen and Wagenknecht 2016; Ide et al. 2016; Krause et al. 2019), larger numbers of morphogens (Diego et al. 2018; Scholes et al. 2019), and a host of other generalizations that explore Turing’s basic insight regarding diffusion-driven pattern formation in increasingly complicated settings; see (Krause et al. 2021) for a broad review. Such extensions relax assumptions that Turing originally made, and add nuance to the core idea of a diffusion-driven instability leading to pattern formation.

One such assumption is the idea that reaction–diffusion processes ‘pre-pattern’ morphogens, which then influence cells downstream to induce changes in cell fate and, hence, spatial organization of tissue structure. The role of spatial heterogeneity in reaction–diffusion systems has been explored extensively in recent years (Maini 1995; Page et al. 2003, 2005; Green and Sharpe 2015; Krause et al. 2018, 2020), plausibly capturing hierarchical pattern formation observed experimentally. There is an inherent separation of timescales needed to justify such an approach to hierarchical pattern formation, in turn motivating studies where this assumption is relaxed with instead tissue restructuring and morphogen dynamics occurring concomitantly on similar timescales in development.

Further related to timescales of reaction–diffusion signalling and tissue restructuring is the role of domain growth, which has also been heavily studied (Crampin et al. 1999, 2002a, b; Plaza et al. 2004; Van Gorder et al. 2021). It is likely that domain growth and restructuring are not downstream of morphogen patterning in many cases, but concomitant processes instead (Boehm et al. 2010). One major insight from these models incorporating domain growth is some amelioration of robustness problems inherent to Turing-type patterning, whereby marginally different initial conditions may evolve to quantitatively different numbers of pattern elements (Maini et al. 2012). Crampin et al. (1999) showed that domain growth could instead lead to a predictable sequence of spike-doubling in 1D reaction–diffusion models, and Ueda and Nishiura (2012) and others have confirmed that this spike-doubling phenomena occurs in generic 1D systems in certain circumstances, such as when the growth is sufficiently slow. While these studies have given important insights into how growth impacts pattern formation, they all consider cases where the domain evolution is explicitly prescribed, and hence cannot account for the feedback between signalling and domain evolution.

There is also a robust chemical setting exploring reaction–diffusion patterning in analogues of growing domains using the photosensitive CDIMA reaction and related chemical systems; see (Konow et al. 2021) for a recent review. Liu et al. (2022) explored a model of this reaction system in terms of a ‘wave of competence’ to pattern formation, and made comparisons to its use as a model of a growing domain. One key point raised in the work by Liu et al. (2022) was that the boundary conditions in such a model likely do not correspond to a moving boundary with simple Neumann or Dirichlet conditions, but possibly something more intricate involving the current concentration on either side of the system.

Following the original formulation of local domain growth given by Crampin et al. (1999), several authors have considered models of concentration-dependent growth (Dillon and Othmer 1999; Neville et al. 2006; Baker and Maini 2007; Seirin Lee et al. 2011b). These studies highlighted a number of important issues in modelling the feedback between domain growth and signalling dynamics, notably the importance of dilution of the domain impacting the structure of pattern elements, as well as the importance of careful constitutive choices made in understanding the complex interplay between domain growth and pattern formation. In most of these studies, the focus was primarily on the slow-growth regime that was qualitatively comparable to prescribed growth scenarios, with only small apparent impacts of concentration-dependence on the overall domain evolution in comparison to prescribed growth scenarios. Neville et al. (2006), following work by Ward and King (1997), did explore faster growth regimes, implicating dilution effects in impacting concentration profiles and, hence, leading to more complex interactions. We also mention that Seirin Lee et al. (2011b) explored the impact of gene-expression time delays in concentration-dependent growth dynamics, which had a nontrivial impact on the timescales and ability for systems to admit spatial patterns.

There is also a large literature developing numerical methods for these kinds of PDE models. In more than one spatial dimension in particular, there are several different choices of numerical approach for growing domains including moving finite-element meshes (Barreira et al. 2011; Dziuk and Elliott 2013), phase-field approaches (Tauriello and Koumoutsakos 2013; Tam and Simpson 2022), and arbitrary Lagrangian–Eulerian frameworks (MacKenzie et al. 2021). Outside of the applications in pattern formation and morphogenesis, there is a growing literature on concentration-dependent domain growth and restructuring in oncology, cell polarity, chemotaxis, and other areas (Chen and Lowengrub 2014; MacDonald et al. 2016).

Another common approach for concentration-dependent growth is to consider models of Stefan-like moving boundary problems, which depend on the local concentrations at the boundary (Yihong and Lin 2010; Sharma and Morgan 2016; Hadeler 2016; Bao et al. 2018; El-Hachem et al. 2019; Sharma et al. 2021; Murphy et al. 2021; Jepson et al. 2022). Such models have been studied intensively in terms of existence theory, travelling waves, and their applications in ecology, epidemiology, and the spreading of cells in developmental and oncological settings. More recently, such models have been explored in terms of reaction–diffusion patterning of a moving boundary (Tam and Simpson 2022), reminiscent of work on wave-initiated patterning (Myerscough and Murray 1992; Krause and Van Gorder 2020) but with the importance that the domain is not fixed. In some cases these moving boundary problems can be seen as a special case of highly localized domain evolution, as shown in the apical growth case of Crampin et al. (2002b), though it is not always the case that one can find an equivalence between these two classes of models, especially in two or more spatial dimensions.

At the tissue-scale, all of the models above use PDE-based models derived from conservation of mass and focus on the morphogen dynamics, while tending to discount the cells themselves (noting the exceptions that Ward and King (1997); Neville et al. (2006) do discuss the importance of mitosis and cell death in these interactions). In comparison, there is now a large computational literature using cell-based and tissue-scale models from other formalisms, such as vertex-based and cellular-Potts models (Osborne et al. 2017; Sharpe 2017; Metzcar et al. 2019; Fletcher and Osborne 2022). Section 7 of Groves et al. (2020) provides an overview of concentration-dependent growth models of the morphogen Shh, as well as some discussion of how likely it is that patterning and growth are concomitant processes. These models, and particularly approaches fitting them to data about cell movement and tissue morphogenesis such as Spiess et al. (2022), are valuable for matching simpler theoretical insights regarding domain restructuring and morphogen signalling.

Here, we consider a simple framework of concentration-dependent growth within the more classical PDE-based approaches in order to elucidate basic theoretical properties of feedbacks between domain evolution and morphogen patterning. We follow the ideas of Crampin et al. (1999), Baker and Maini (2007), and Seirin Lee et al. (2011b), where we model a local volume element of the tissue as either expanding or contracting in time, depending on morphogen concentration. We present a version of such a model for a general *N*-dimensional manifold in Sect. 2, as in the prescribed growth models of Plaza et al. (2004) and Van Gorder et al. (2021), showing why local volume evolution is insufficient to fully characterize the evolution of either the domain or the concentrations for $N>1$, necessitating further constitutive assumptions. In the 1D case, we use the framework of Van Gorder et al. (2021) and Van Gorder (2020) to perform a linear stability analysis of homogeneous base states in Sect. 3, deriving an extra term arising due to concentration-dependence. We numerically explore the 1D model in a variety of less-explored regimes in Sect. 4, namely those involving domain contraction and rapid local evolution, where dilution leads to complex interplays of pattern structure and domain evolution, mediated both by the size of the domain but also by the impact of highly localized dilution. We then pose a simple constitutive model for *N*-dimensional manifolds that is both simple and plausible. We explore this model numerically in 2D planar geometries, demonstrating simple but important lessons in the constitutive choices made, as well as striking behaviours of patterns even in simple concentration-dependent settings. We close with a discussion of our results in Sect. 6, highlighting in particular the rich and unexplored dynamics of these systems. One important insight described here is the tractability of the 1D system to mathematical exploration and how this contrasts the difficulties in both modelling and analysis for any higher-dimensional generalizations.

## A Simple Model of Concentration-Dependent Domain Evolution

We formulate a general approach to modelling morphogen-dependent growth for *N*-dimensional manifold domains with boundaries, and describe concrete versions of this in one-dimensional and planar domains. We follow the general notation from Krause et al. (2019) and Van Gorder et al. (2021), though refer to Crampin et al. (1999), Crampin et al. (2002b), and Seirin Lee et al. (2011a) for an equivalent discussion using different terminology/notation.

We consider *m* morphogen concentrations $\varvec{u} = (u_1,u_2,\dots ,u_m)$ on a compact evolving domain $\Omega (t) \subset {\mathbb {R}}^N$, focusing on the cases $m=1$ and $m=2$. We assume that this domain is bounded by a sufficiently smooth simple closed hypersurface $\partial \Omega (t)$ for all time *t*. By considering conservation of mass in a non-dimensionalized setting and moving to a Lagrangian frame, we find that these morphogens satisfy

1 $$\begin{aligned} \frac{\partial \varvec{u}}{\partial t} = \varvec{D}\nabla ^2_{\Omega (t)}{} \varvec{u} - \varvec{u}\left( \nabla _{\Omega (t)}{} \cdot \varvec{a}\right) + \varvec{f}(\varvec{u}), \end{aligned}$$

where $\varvec{D} = \text {diag}(D_1, D_2, \dots D_m)\in {\mathbb {R}}^{m\times m}$ is a diagonal matrix of diffusion coefficients, $\varvec{f}(\varvec{u})\in {\mathbb {R}}^m$ the vector of reaction kinetics, $\varvec{a}\in {\mathbb {R}}^N$ the material flow defining the domain evolution, and $\nabla ^2_{\Omega (t)}{}$ and $\nabla _{\Omega (t)}{} \cdot$ are, respectively, the Laplace–Beltrami^1^ and divergence operators on the domain $\Omega (t)$. The divergence of $\varvec{a}$ represents a dilution of concentration during domain growth. We will assume no-flux conditions at any domain boundary, and specify initial concentrations $\varvec{u}(0,\varvec{X})$ where $\varvec{X} = (X_1, X_2,\dots ,X_n)^T \in \Omega (0)$ are the initial Lagrangian coordinates. We will write the spatial derivatives in terms of a metric tensor $\varvec{G} = (G^{ij})$, written in the Lagrangian coordinates $\varvec{X}$, and denote the inverse by $\varvec{H} = \varvec{G}^{-1}$. In these coordinates, we have that the Laplace–Beltrami operator can be written as

2 $$\begin{aligned} \nabla ^2_{\Omega (t)}{} u = \frac{1}{\sqrt{|\det \varvec{G}|}}\sum _{i,j=1}^n \frac{\partial }{\partial X_i}\left( \sqrt{|\det \varvec{G}|} H^{ij} \frac{\partial u}{\partial X_j}\right) , \end{aligned}$$

and the dilution coefficient as

3 $$\begin{aligned} \nabla _{\Omega (t)}{} \cdot \varvec{a} = \frac{\partial }{\partial t}\log \left( \sqrt{|\det \varvec{G}|}\right) . \end{aligned}$$

We note that $\mu = \sqrt{|\det \varvec{G}|}$ is the coefficient of the volume form, and in Lagrangian coordinates $\varvec{X}$ can be thought of as the local expansion or contraction of the domain. We will assume that the domain evolves according to a local isotropic expansion or contraction at a rate $S(t,\varvec{u})$, that is,

4 $$\begin{aligned} \nabla _{\Omega (t)}{} \cdot \varvec{a} = \frac{\partial }{\partial t}\log (\mu )= S(t,\varvec{u}). \end{aligned}$$

As we are mapping from the initial domain $\Omega (0)$ to $\Omega (t)$, we take $\mu (0,\varvec{X}) =1$. Note that, for ease of presentation, we are taking for granted that there is a global coordinate system used to express $\varvec{G}$ locally, though in principle there is no obstruction to defining the transport terms in more complicated situations that require multiple coordinate charts, etc. We will also assume that the mappings remain sufficiently smooth, so that, in particular, $\sqrt{|\det \varvec{G}|}>0$ for all time *t*. We assume that the concentrations satisfy Neumann boundary conditions along all boundaries throughout this work. We will primarily be interested in cases where *S* depends only on the concentrations, rather than explicitly on time *t*, but we include this dependence to recover well-studied models of prescribed growth with $S=S(t)$.

### One-Dimensional Model

As noted by Seirin Lee et al. (2011a), in one spatial dimension Eqs. (1)–(4) can be made into a closed system by fixing a stationary point in the Eulerian frame (and the choice of such a point does not influence the dynamics). We can see this by considering the Eulerian coordinate $x(t)\in \Omega (t)$, which is related to the Lagrangian $X\in \Omega (0)=[0,L]$ (for some initial domain length $L>0$) by the scalar metric^2^

5 $$\begin{aligned} \mu (t,X) = \sqrt{|G(t,X)|} = \frac{\partial x(t)}{\partial X}. \end{aligned}$$

One can then integrate (5) to determine how material points move. The reaction–diffusion system (1) then takes the form

6 $$\begin{aligned} \frac{\partial \varvec{u}}{\partial t} = \frac{\varvec{D}}{\mu } \frac{\partial }{\partial X}\left( \frac{1}{\mu } \frac{\partial \varvec{u}}{\partial X} \right) - \varvec{u}S(t,\varvec{u}) + \varvec{f}(\varvec{u}), \end{aligned}$$

with the growth dynamics given by

7 $$\begin{aligned} \frac{\partial }{\partial t}\log (\mu ) = S(t,\varvec{u}). \end{aligned}$$

We can integrate (7) and use (5) to find that material points are given by,

8 $$\begin{aligned} x(t,X) = \int _0^X\mu (t,y)\mathop {}\!\textrm{d}{y} = \int _0^X \exp \left( \int _0^t S(s,\varvec{u}(s,y)) \mathop {}\!\textrm{d}{s}\right) \mathop {}\!\textrm{d}{y}, \end{aligned}$$

where we are fixing the Eulerian domain to have the same zero, i.e. $x(t,0)=0$, so that $\Omega (t) = [0, x(t,L)]$. Equations (6)–(7) can then be solved on the fixed Lagrangian coordinates, $X \in [0,L]$, (independently of fixing an Eulerian point) and visualized on the Eulerian domain given by (8). While in theory all of the dynamics is encoded in the Lagrangian equations alone, in practice the Eulerian domain is also computed to help simulate these equations, as they can become numerically ill-posed over moderate timescales on the Lagrangian domain due to rapid separation or clustering of material points—see Sect. 4 for details.

### $N>1$-Dimensional Model

In two spatial dimensions, Eqs. (1)–(4) do not provide a closed system that uniquely determines the concentrations and domain evolution. While (4) can be solved for $\mu$, this does not determine the full metric tensor $\varvec{G}$, which is needed to interpret the Laplace–Beltrami operator in (1), as well as to determine how material points move. Equivalently, there is not a unique flow $\varvec{a}$ which satisfies (3). Here, we will make some of the simplest possible constitutive assumptions in order to derive a model on a compact, simply connected *N*-dimensional manifold. We then focus our analysis on a two-dimensional planar domain as the simplest example of this. Our assumptions will be of a kinematic nature, and will neglect a more detailed mechanical consideration of growing or deforming tissue.

We assume that the local flow is irrotational as a simple constitutive constraint, together with the assumption that the flow has no tangential component at the boundaries of the domain. These two constraints are fully consistent with the assumption that the flow is generated *only* by point sources of density scaling with *S* and that cells constituting the domain cannot move along the edges of the domain (Sections 2.4, 2.5, Batchelor (1967)).

With these constitutive constraints, and given sufficient smoothness, we have by the Helmholtz Decomposition Theorem that the flow $\varvec{a}$ is a conservative vector field, so that there is some scalar potential $\phi$ such that $\varvec{a} = \nabla _{\Omega (t)}{}\phi$. Combining this assumption with (4), we find that $\phi$ satisfies a Poisson equation on $\Omega (t)$ given by

9 $$\begin{aligned} \nabla ^2_{\Omega (t)}{}\phi = S(t,\varvec{u}) \end{aligned}$$

and, by using (2), we can write this as an equation on the Lagrangian domain $\Omega (0)$ in terms of the metric tensor $\varvec{G}$ in the coordinates $\varvec{X}$. To complete the specification of $\varvec{a}$, we need a suitable boundary condition for (9). Given the further assumption that the flow at domain boundaries is purely normal, i.e. $\varvec{a}\cdot \varvec{t}=0$ on $\partial \Omega (t)$ for any unit tangent vector $\varvec{t}$, we have $\varvec{t}\cdot \nabla _{\Omega (t)}{}\phi =0$, so that $\phi$ is constant along $\partial \Omega (t)$. As the potential $\phi$ is only defined up to a spatially uniform function of time, without loss of generality we set

10 $$\begin{aligned} \phi = 0, ~~~ \varvec{x} \in \partial \Omega (t) \end{aligned}$$

where $\partial \Omega (t)$ denotes the boundary. Furthermore, $\partial \Omega (t)$ will be determined by the material points from $\partial \Omega (0)$, so we can interpret this as a homogeneous Dirichlet condition on $\phi$ in the Lagrangian frame as well.

Finally, in order to relate Eulerian and Lagrangian points, we can write out the flow as

11 $$\begin{aligned} \frac{\partial \varvec{x}}{\partial t} = \varvec{a} = \nabla _{\Omega (t)}{}\phi . \end{aligned}$$

In the $N=2$ planar setting, this can be expanded as

12 $$\begin{aligned} \frac{\partial x_1}{\partial t}&= \phi _{X_1}\left( H^{11}\frac{\partial x_1}{\partial X_1} + H^{12}\frac{\partial x_1}{\partial X_2}\right) + \phi _{X_2}\left( H^{21}\frac{\partial x_1}{\partial X_1} + H^{22}\frac{\partial x_1}{\partial X_2}\right) , \end{aligned}$$

13 $$\begin{aligned} \frac{\partial x_2}{\partial t}&= \phi _{X_1}\left( H^{11}\frac{\partial x_2}{\partial X_1} + H^{12}\frac{\partial x_2}{\partial X_2}\right) + \phi _{X_2}\left( H^{21}\frac{\partial x_2}{\partial X_1} + H^{22}\frac{\partial x_2}{\partial X_2}\right) , \end{aligned}$$

where the subscripts denote partial derivatives and $\varvec{x} = (x_1,x_2)^T$ are the Eulerian coordinates. Equations (12)–(13) consist of a hyperbolic system of first-order partial differential equations, so we do not prescribe boundary conditions and instead compute the flow of material points directly. We can write the inverse metric tensor, $\varvec{H}$, in terms of derivatives of the Eulerian coordinates as

14 $$\begin{aligned} H^{11} = \frac{1}{\mu ^2}\left| \frac{\partial \varvec{x}}{\partial X_2}\right| ^2, \quad H^{12} = H^{21} = -\frac{1}{\mu ^2}\frac{\partial \varvec{x}}{\partial X_1} \cdot \frac{\partial \varvec{x}}{\partial X_2},\quad H^{22} = \frac{1}{\mu ^2}\left| \frac{\partial \varvec{x}}{\partial X_1}\right| ^2, \end{aligned}$$

and the coefficient of the volume form as

15 $$\begin{aligned} \mu = \sqrt{ \left| {\frac{\partial \varvec{x}}{\partial X_1}} \right| ^2\left| {\frac{\partial \varvec{x}}{\partial X_2}} \right| ^2 - \left( \frac{\partial \varvec{x}}{\partial X_1}\cdot \frac{\partial \varvec{x}}{\partial X_2}\right) ^2}. \end{aligned}$$

Equations (9)-(15) form a closed system for the Eulerian coordinates as functions of the Lagrangian coordinates, and hence components of the metric tensor, once values of $\varvec{u}$ are provided to determine $S(t,\varvec{u})$. That is, Eqs. (1) and (9)-(15) form a closed system for the evolution of concentrations on an evolving domain, written entirely in the Lagrangian reference frame. While we will only consider finite planar 2D domains for numerical simplicity, our formulation works for a general manifold with boundary.

## Linear Instability Analysis

Here, we provide a linear instability analysis of homogeneous base states (generalizing homogeneous equilibria) for (1)-(4) in the case of $m=2$ and $N=1$, describing the obstacles to generalizing this analysis at the end. Due to the non-autonomous nature of an evolving domain, there are different choices for defining a notion of a base state in order to study linear instabilities (Van Gorder et al. 2021). Here, we use a natural generalization of a homogeneous base state $(\varvec{u}^*(t), \mu ^*(t))$, which evolves as

16 $$\begin{aligned} \frac{\textrm{d} \mu ^*}{\textrm{d} t}= & {} S(t,\varvec{u}^*)\mu ^*, \end{aligned}$$

17 $$\begin{aligned} \frac{\textrm{d} \varvec{u}^*}{\textrm{d} t}= & {} - S(t,\varvec{u}^*)\varvec{u}^* + \varvec{f}(\varvec{u}^*), \end{aligned}$$

where we use $\varvec{f} = (f,g)^T$ as a vector of reaction kinetics. As in Van Gorder et al. (2021), we take $\varvec{u}^*(0)$ to satisfy $\varvec{f}(\varvec{u}^*(0))=\varvec{0}$ to agree with the linear stability analysis on static manifolds, and take $\varvec{\mu }^*(0)=1$. We perturb the system (1)-(4) by writing^3^$\varvec{u} = \varvec{u}^*(t) + \varepsilon \varvec{U}^*(t,\varvec{x})$ and $\mu = \mu ^*(t) + \varepsilon \nu (t,\varvec{x})$ for $|\varepsilon | \ll 1$. Substituting these into (6)-(7) and retaining only terms up to order $\varepsilon$, we find that the perturbations satisfy

18 $$\begin{aligned} \frac{\partial \nu }{\partial t}= & {} S(t,\varvec{u}^*)\nu + \left( \nabla _{\varvec{u}}S \cdot \varvec{U}\right) , \end{aligned}$$

19 $$\begin{aligned} \frac{\partial \varvec{U}}{\partial t}= & {} \frac{\varvec{D}}{(\mu ^*)^2} \frac{\partial ^2 \varvec{U}}{\partial X^2} - S(t, \varvec{u}^*)\varvec{U} - \varvec{K}\varvec{U}+ \varvec{J}\varvec{U}, \end{aligned}$$

where we define

$$\begin{aligned} (\nabla _{\varvec{u}}S)_i = \frac{\partial S}{\partial u_i}(t,\varvec{u}^*), \quad K_{ij} = \frac{\partial S}{\partial u_j}(t,\varvec{u}^*)u_i^*, \quad J_{ij} = \frac{\partial f_i}{\partial u_j}(\varvec{u}^*), \quad i,j=1,2. \end{aligned}$$

We note that (19) does not depend on $\nu$, as terms involving $\nu$ will be multiplied by gradients of $\varvec{u}^*$, which will vanish as these are spatially homogeneous. Hence, linear perturbations in the manifold evolution do not play a role in the stability of homogeneous equilibria. Therefore, we can neglect (18), and instead study just the linear system (19) subject to the base states defined by (16)-(17). We can also see that (17) does not depend on $\mu ^*$, so that we can directly solve (16) in terms of the function $\varvec{u}^*$. If *S* does not depend explicitly on time *t*, we find that the base state of the manifold, and its growth or contraction, is spatially uniform, in particular with the base state of the concentrations evolving autonomously via Eq. (17).

This system can be solved via the method introduced in Van Gorder et al. (2021). More directly, in Van Gorder (2020) the authors study linear systems of exactly the form (19) if we define the linearized kinetics as $\varvec{M} = {\varvec{J}} - \varvec{K} - S(t,\varvec{u}^*)\varvec{I}$, where $\varvec{I}$ is the two-by-two identity matrix, and write $\mu ^*(t) = \exp (\int _0^tS(s,\varvec{u}^*(s))\mathop {}\!\textrm{d}{s})$. The key insight is that the time-dependence of the Laplace–Beltrami operator can be completely factored out, so that one can take a Lagrangian decomposition of $\varvec{U}$:

20 $$\begin{aligned} \varvec{U}(t,{X}) = \sum _{k=0}^\infty \psi _k({X})\varvec{V}_k(t), \quad \frac{\partial ^2 \varvec{\psi } _k}{\partial X^2} = {\rho _k}\psi _k(\varvec{X}) , \end{aligned}$$

where the constant spatial eigenvalues ${\rho _k} = (k \pi /L)^2$ correspond to the standard Laplacian on an interval [0, *L*] with associated eigenfunctions $\psi _k(X) = \cos (k \pi x/L)$. Perturbations then grow according to the non-autonomous ODE system

21 $$\begin{aligned} \frac{\partial \varvec{V}_k}{\partial t} = -\rho _k(\mu ^*(t))^{-2}\varvec{D}\varvec{V}_k + \varvec{M}(t)\varvec{V}_k. \end{aligned}$$

Theorem 2.1 of Van Gorder (2020) then gives a differential inequality that implies linear instability of a given mode over an interval of time, which we state here in our notation.

### Theorem 1

(Van Gorder 2020) Let ${\mathcal {I}}_k\subset [0,\infty )$, and assume that $\varvec{M}$ is not diagonal for any $t \in {\mathcal {I}}_k$. Then, the perturbations associated with the *k*th mode, $\varvec{V}_k$, grow exponentially if

22 $$\begin{aligned}{} & {} \det (\varvec{M}(t))-\frac{D_1M_{11}(t) + D_2M_{22}(t)}{(\mu ^*(t))^2}\rho _k + \frac{D_1D_2}{(\mu ^*(t))^4}\rho _k^2\nonumber \\{} & {} < \max \left\{ M_{12}(t)\frac{\text {d} }{\text {d} t}\left( \frac{M_{11}(t)(\mu ^*(t))^2-D_1\rho _k}{M_{12}(t)(\mu ^*(t))^2} \right) , M_{21}(t)\frac{\text {d} }{\text {d} t}\left( \frac{M_{22}(t)(\mu ^*(t))^2-D_2\rho _k}{M_{21}(t)(\mu ^*(t))^2} \right) \right\} \end{aligned}$$

is satisfied for all $t \in {\mathcal {I}}_k$.

This criterion gives a local-in-time condition for perturbations to be linearly unstable. Of course, pattern formation requires such instabilities to grow to sufficient amplitudes to be observable against the possibly complex behaviour of the spatially uniform base state given by $\varvec{u}^*(t)$. In particular, sufficiently rapid variations of $\varvec{u}^*$ in time can prevent patterns from forming due to the interval of instability associated with a particular mode, ${\mathcal {I}}_k$, being too small. Typically when patterns do form, they may stay within the linearly predicted regime but deviate from the maximally unstable mode due to nonlinear effects such as peak splitting (Crampin et al. 1999; Ueda and Nishiura 2012). We do note that a new term appears in our instability condition due to concentration-dependence, namely $\varvec{K}$. One can show that, for sufficiently slow-growth regimes, this instability criterion (and a stability condition for $k=0$) reduce to a quasi-static correction of the usual Turing conditions, which was computed in a different manner by Madzvamuse et al. (2010).

Additionally, in the concentration-dependent setting, these linear stability results are even more limited as they only hold for short times, when the growth is effectively uniform. Local growth of the domain can lead to substantial differences from such an assumption due to nonlinear effects (e.g. local dilution interacting with kinetics). Finally, this analysis cannot be repeated for $N>1$-dimensional domains, as was done for coordinate-dilational growth in Van Gorder et al. (2021), because our constitutive assumptions for the flow do not allow the eigenfunctions to be separable due to non-uniformity in the growth even for constant *S*, as shown in Fig. 7.

## One-Dimensional Numerical Results

We now explore the 1D model numerically, seeking to examine the impact of concentration-dependent growth compared to explicitly pre-defined forms of growth on the pattern-forming behaviour of classical reaction–diffusion models. In particular, the dilation rate *S* will be specified on a case by case basis below. Furthermore, writing $\varvec{u} = (u,v)$ and $\varvec{f} = (f,g)$, we will consider the Schnakenberg kinetics (Schnakenberg 1979; Murray 2004):

23 $$\begin{aligned} f(u,v) = a - u + u^2v, \quad g(u,v) = b - u^2v, \end{aligned}$$

the Gierer–Meinhardt kinetics (Gierer and Meinhardt 1972):

24 $$\begin{aligned} f(u,v) = a - \frac{u^2}{v} -bu, \quad g(u,v) = u^2-cv, \end{aligned}$$

and the FitzHugh–Nagumo kinetics (FitzHugh 1955; Nagumo et al. 1962):

25 $$\begin{aligned} f(u,v) = c\left( u-\frac{u^3}{3}+v-i_0\right) , \quad g(u,v) = \frac{(a-u-bv)}{c}. \end{aligned}$$

In all cases we consider positive parameters, i.e. $a,b,c,i_0>0$.

We will also demonstrate the impact of concentration-dependent growth on the dynamics of scalar reaction–diffusion models using the (nondimensionalized) logistic (Fisher 1937; Murray 2007) and bistable (Chafee and Infante 1974; Keener 2021) kinetics, respectively, given by

26 $$\begin{aligned} f(u)= & {} u(1-u), \end{aligned}$$

27 $$\begin{aligned} f(u)= & {} u(1-u^2). \end{aligned}$$

We simulate (6)-(7) using a simple method-of-lines finite-difference scheme in MATLAB. In particular, the diffusion term is discretized as,

28 $$\begin{aligned} \frac{1}{\mu }\frac{\partial }{\partial X}\left( \frac{1}{\mu }\frac{\partial u}{\partial X} \right)\approx & {} \frac{1}{2\mu _i}\left( \frac{1}{\mu _i}(u_{i+1}+u_{i-1}-2u_i)+\frac{1}{\mu _{i+1}}(u_{i+1}-u_i)\right. \nonumber \\ {}{} & {} \quad \left. +\frac{1}{\mu _{i-1}}(u_{i-1}-u_i)\right) , \end{aligned}$$

where $u_i$ and $\mu _i$ represent these variables at the *i*th grid point. The resulting system of ODEs is integrated in time using the MATLAB function ode15s, which implements a variable-step, variable-order solver (Shampine and Reichelt 1997). Relative and absolute tolerances are taken to be $10^{-11}$ and, unless otherwise mentioned, $N_s=10^4$ equispaced grid points are used. For the kinetics (23)-(25), initial data are taken as a perturbation of the homogeneous steady state of the form $u(0,x) = u^*(1+\eta (x)), v(0,x) = v^*(1+\xi (x))$, where $\eta$ and $\xi$ are independently and identically normally distributed random variables with zero mean and variance $10^{-2}$. We note that the kinetics (25) may have up to three homogeneous equilibria, but we only consider parameters where there is one real equilibrium.

Due to the rapid (often exponential) separation of material points, each simulation is broken up into several iterations where, at the end of one iteration, the final Eulerian domain given by (8) becomes the new Lagrangian domain. This new computational domain is then uniformly discretized, and $\mu$ is set to unity throughout the domain. Interpolating the morphogen concentrations onto this new computational domain, this overall procedure ensures relative uniformity of the mesh compared to a single fixed Lagrangian domain. Numerical solution of the recast Lagrangian equations proceeds until the next discrete epoch, at which point the computational domain is once again redefined and remeshed. Here, we choose the iteration size in an ad hoc nature for each considered example, though in principle one could utilize some metric of mesh quality to adaptively select these lengths. Refinements of this iteration procedure had no impact on any of the reported simulations. Maximum timestep controls and grid point refinements were also used to check convergence in select simulations. Additionally, a COMSOL LiveLink implementation was also implemented for the 1D model, incorporating mesh refinement between iterations, using a comparable method to that detailed in Sect. 5 for the two-dimensional simulations. These different numerical methods gave quantitatively comparable solutions on sufficiently fine meshes. All code and associated documentation can be found at Krause et al. (2022).

### Travelling Waves in Diffusive Logistic Equations

**Fig. 1:**
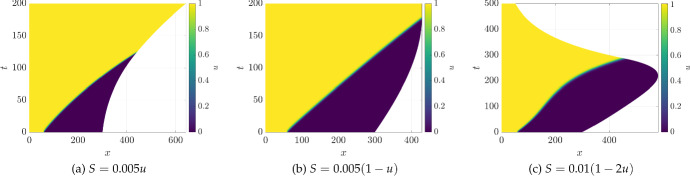
(Color Figure Online) Values of *u* from 1D simulations of the logistic kinetics (26) under different growth scenarios. In all simulations, the initial domain length is $L=30$, with $D_1=1$. The initial condition is taken as $u(0,x) = (1+\tanh (L/5-x))/2$, so that the leftmost $20\%$ of the domain has the initial value $u \approx 1$ and the rest has the value $u \approx 0$

To begin our exploration of concentration-dependent growth, we first consider the scalar case of $m=1$ and the logistic growth model given by the kinetics (26). This model has been a paradigm of emergent travelling waves due to the interactions of diffusion and nonlinearity; see Chapter 13 of Murray (2007) for an overview of this and related models. We save a detailed discussion of wave-type behaviour in these models for future work, giving instead just three examples of how concentration-dependent growth can change the structure of the typical constant-speed travelling wave observed in this model.

We show these three examples in Fig. 1. For panel (a), we observe exponential growth, though at an increasing rate as the region for which $S(u) \approx 0.005$ grows as the wave travels across the domain. After $t \approx 120$ time units, the domain growth saturates to a constant exponential rate. In contrast, panel (b) shows an example where the region of positive growth decreases as the wave advances, eventually halting around $t \approx 180$. We remark that this solution is approaching (exponentially as *u* tends to 1) a homogeneous equilibrium of the model on a fix domain size, where both *u* and $\mu$ reach a fixed value in time. Finally, panel (c) gives the most exotic dynamics, where the domain is contracting whenever $u \approx 1$, and the domain is growing whenever $u \approx 0$. This leads to a transient period of growth as the wave moves, changing the proportion of the domain that is growing, until growth ceases and the domain begins contracting. Once the entire domain has approximately reached the $u=1$ steady state, it henceforth contracts at a fixed exponential rate.

**Fig. 2:**
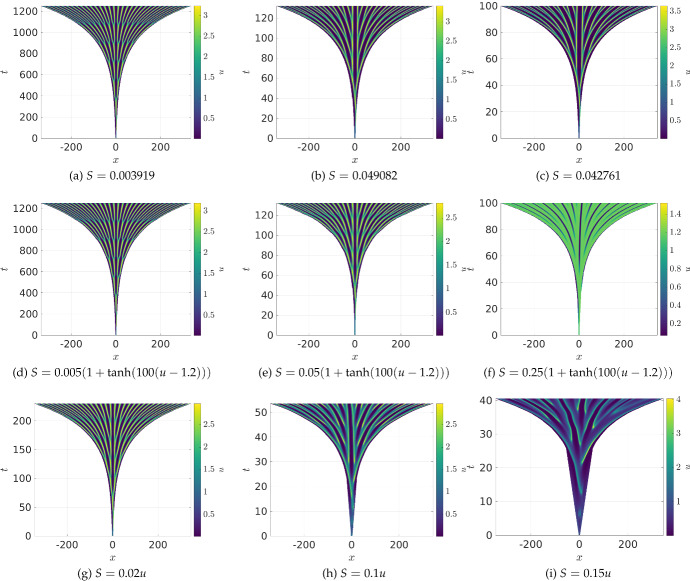
(Color Figure Online) Values of *u* from 1D simulations of the Schnakenberg kinetics (23) under different growth scenarios. In all simulations the initial domain length is $L=5$, with $a=0.01$, $b = 1.1$, $D_1=1$, and $D_2=40$. In (**d**)–(**i**), we show *u*-dependent growth, whereas in (**a**)–(**c**) we show uniform exponential domain growth. Timescales are set so that all domains grew to $\approx 670$. Uniform growth rates were used in (**a**)–(**c**) to match the corresponding domain lengths and timescales in (**d**)–(**f**), so that the final simulation time and domain sizes are identical

There is a nontrivial impact of the growth on the apparent speed of the wave front, which is consistent with other models of uniform growth on travelling waves (Landman et al. 2003). One has to be careful in defining a wave speed in this case, in part due to how one defines distances in evolving domains. This highlights one important feature of choosing $x = X = 0$ as a fixed point in the Eulerian–Lagrangian mapping, as it makes the rightmost point move the most, though in reality the domain growth and contraction is occurring locally throughout the domain. We leave further exploration of these ideas to future work and, instead, focus on pattern formation in the rest of the paper. In all subsequent 1D figures, we will instead fix the midpoint of the domain in our visualizations.

### Pattern Formation in Schnakenberg Systems

**Fig. 3:**
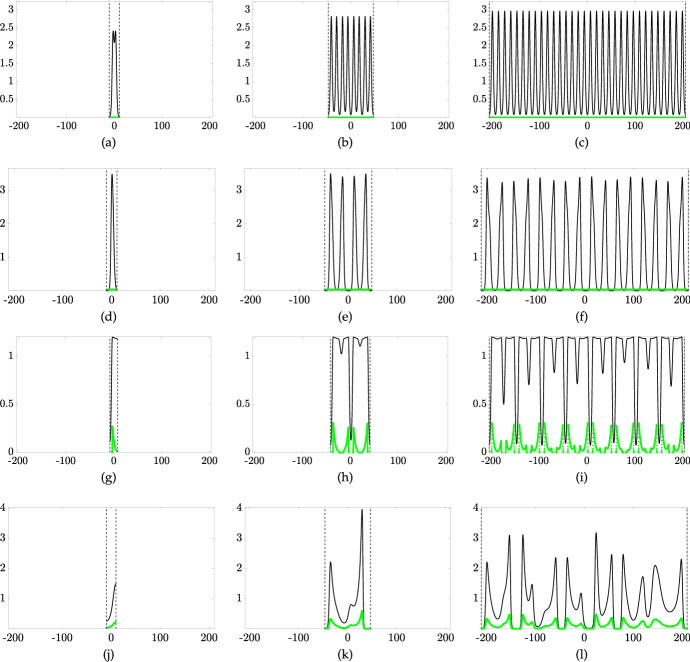
(Color Figure Online) Values of *u* as black curves and *S* as green curves at specific times from the same simulations in Fig. 2. Row (**a**)–(**c**) corresponds to panel (**a**) in Fig. 2, Row (**d**)–(**f**) corresponds to panel (**c**) in Fig. 2, row (**g**)–(**i**) corresponds to panel (**f**) in Fig. 2, and Row (**j**)–(**l**) corresponds to panel (**i**) in Fig. 2. The first column is taken at 1/3 of the final simulation time, the second at 2/3 of the final simulation time, and the last column at the final simulation time

We give examples of growing domain simulations in Fig. 2 for the Schnakenberg kinetics (23). Panels (a)–(c) are uniformly exponentially growing domains, whereas those in (d)–(f) grow in a ‘thresholded’ manner (that is, regions of space grow approximately where $u>1.2$), and those in (g)–(i) grow at a rate proportional to *u*. The panels are arranged so that the columns moving left to right show increasingly fast growth timescales. Hence, the most drastic impacts of the different kinds of growth can be observed in the last column, where we see in (c) that fast uniform growth has the same qualitative character of peak splitting as for lower growth rates. In contrast, the fast thresholded growth in (f) leads to large regions of *u* that are saturated just beyond the growth threshold, as local dilution decreases the maximum value of *u* in the fastest growing regions. Finally, in (i), we see that the very high growth rates in spikes can lead to a complete breakdown of the more usual spike-doubling behaviour observed at low growth rates, and the insertion of irregular spikes. This is consistent with such behaviours observed for fast uniform growth, which has been described analytically in Ueda and Nishiura (2012) (see also Kolokolnikov et al. (2006) for an alternative view of the underlying mechanism behind such spike-doubling phenomena). However, for the concentration-dependent cases, this deviation from spike-doubling is more pronounced and leads to extremely irregular insertion events. One important observation that was confirmed across many other simulations is that, for sufficiently small growth rates, as long as $S \ge 0$, similar spike-doubling behaviour was typically observed, as seen here in the first column.

**Fig. 4:**
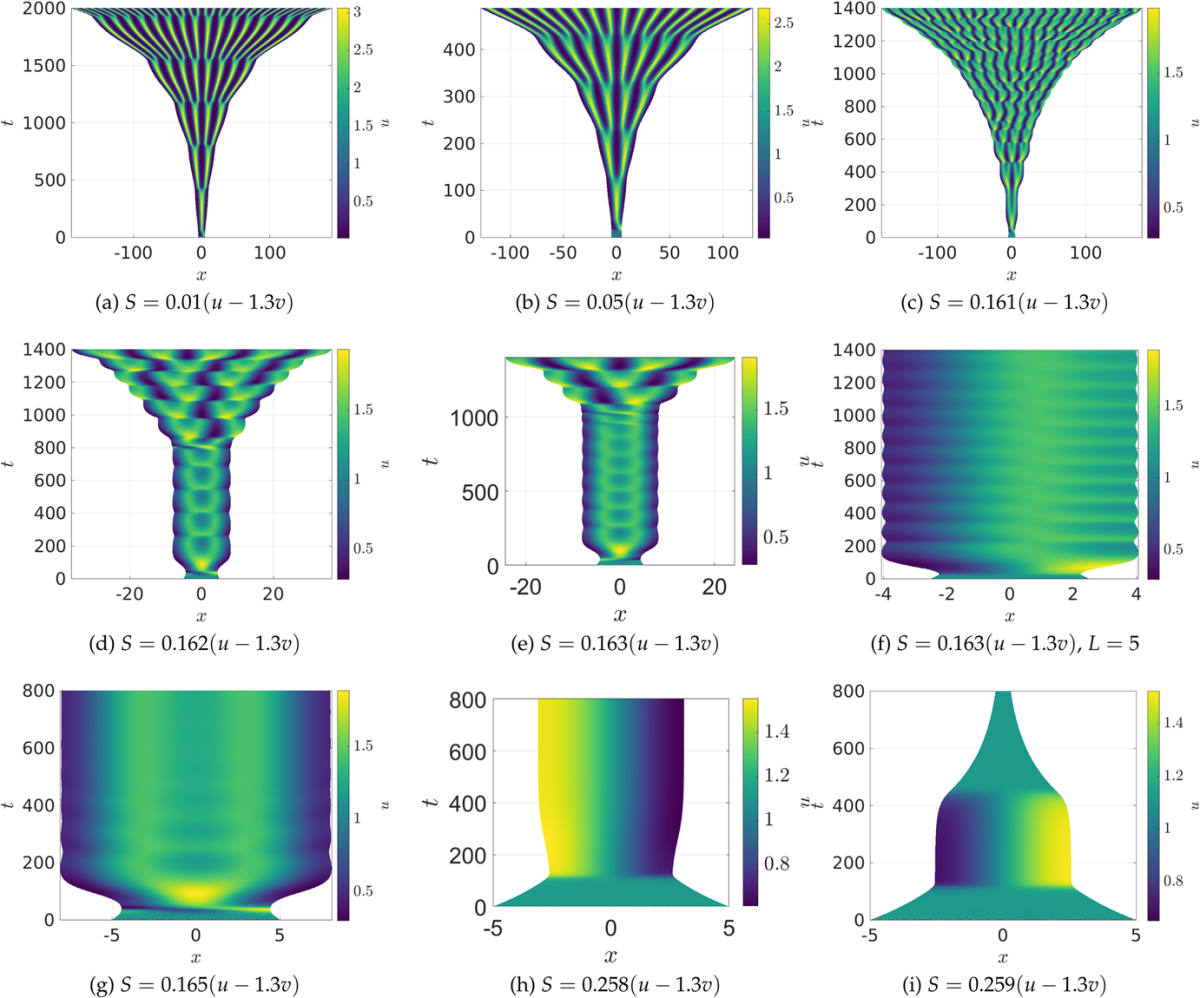
(Color Figure Online) Values of *u* from 1D simulations of the Schnakenberg kinetics (23) under different growth scenarios. In all simulations, the initial domain length is $L=10$, except in (f) where $L=5$, with $a=0.01$, $b = 1.1$, $D_1=1$, and $D_2=40$

In Fig. 3, we plot particular profiles of *u* and *S* from the preceding simulations at selected time points. As detailed in the caption, the first two rows correspond to the slow and fast uniform growth rates, whereas the third row is the fastest thresholded growth scenario, and the fourth is the fastest growth case with $S \propto u$. The columns are, from left to right, at increasing fractions of the total simulation time. While there are some transient differences between uniform growth at different rates (compare (b) with (e)), broadly the same spike-doubling behaviour is observed, and away from insertion events the spikes maintain an approximately uniform spacing, as they do on fixed domains. In contrast, the thresholded growth shown in (g)–(i) initiates new regions not by splitting sharp peaks, but by growing sharp valleys between plateaus of high *u* concentration, with growth localized at the ends of these plateaus. The profiles with $S \propto u$ are far more irregular throughout the simulation, with large spikes appearing and moving away from regions where new but smaller spikes initiate. This rapid movement of large spikes is a complex interplay of *u* and *v* having different regions of localization (approximately out-of-phase), and the nonlinearity inherent in the dilution term.

We now explore a scenario where both domain growth and contraction occur simultaneously. In Fig. 4, we give examples where $S = r(u-1.3v)$ with $r>0$ a constant. The factor of 1.3 present in *S* was chosen as the steady state values satisfy $u^* - 1.3v^*<0$, but integrating this expression for a patterned state generated on a large fixed domain gave a positive net growth rate. Depending on the constant of proportionality and initial domain length *L*, we observe exponential-like (though still complex) growth for $r \le 0.161$ in (a)-(c), oscillations leading eventually to growth for $0.162 \le r \le 0.163$ in (d) and (e), oscillations or transient domain shrinking leading eventually to a fixed domain size for $0.164 \le r \le 0.259$ in (g) and (h), and, finally, domain shrinkage for $r \ge 0.259$ as in (i). Additional simulations within each range of *r* values leads us to believe that, at least for a fixed initial condition and domain size, these qualitatively different regimes can be tuned via the parameter *r*. We did observe different dynamics for $L=5$ where, for $r=0.163$, we see sustained oscillations in panel (f). We confirmed the predictions from panels (f)–(h) appear to be genuine long-time behaviours by simulating these cases over timescales 20 times longer and checking convergence in space and time steps. While these parameters are intentionally chosen to demonstrate this wide variety of behaviour, similar kinds of dynamics were observed for a range of choices of *S*(*u*). One important remark is that while panels (g) and (h) show concentrations and domains approaching a fixed state, the system does not truly reach an equilibrium as the domain will continue expanding and contracting indefinitely. In other words, unlike panel (b) of Fig. 1, the value of $\mu$ given by (7) never reaches an equilibrium.

### Pattern Formation in Gierer–Meinhardt & FitzHugh–Nagumo Systems

**Fig. 5:**
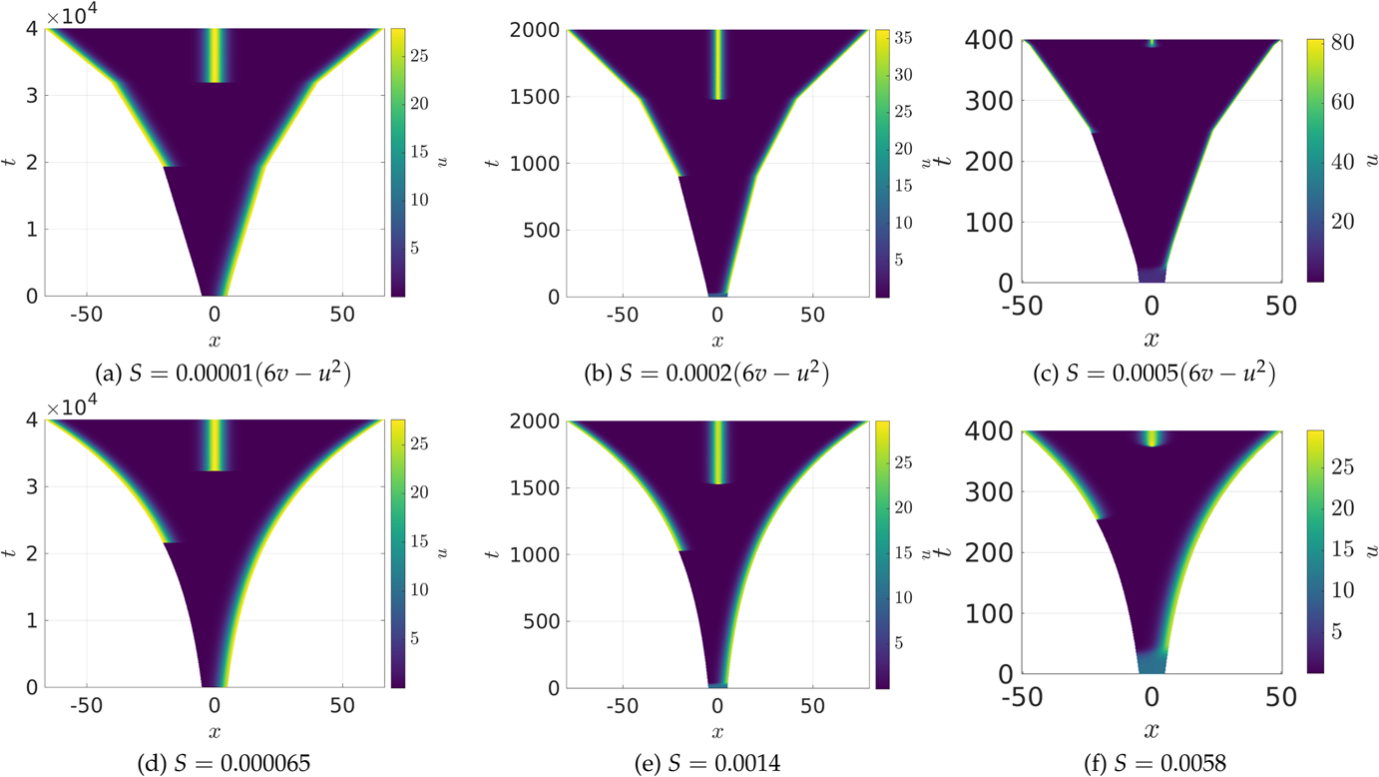
(Color Figure Online) Values of *u* from 1D simulations of the Gierer–Meinhardt kinetics (24) under different growth scenarios. In all simulations the initial domain length is $L=10$, with $a = 0.01$, $b = 0.5$, $c = 5.5$, $D_1=1$ and $D_2=200$. The timescale and growth rates in (a)–(c) are chosen to match those in (e)–(g) so that the final simulation time is on a domain of exactly the same size

We next consider examples of competing growth and contraction using the Gierer–Meinhardt kinetics (24). As opposed to the preceding subsection, these kinetics have the activator *u* in phase with the faster-diffusing inhibitor *v*, so that both species become highly localized in the same spike regions, leading to localized regions of contraction and growth within the domain near spikes. We choose the form $S(u,v) = r(6v-u^2)$ for varying $r>0$ so that overall there is a small net positive growth rate despite large local contraction and growth. The particular choice of this functional form is partially inspired by the form of *g*(*u*, *v*), but, as in the choice of the nonlinearity in Fig. 4, is chosen primarily to illustrate some of the interesting phenomena that can occur in combining growth and contraction.

We plot three examples of this growth in panels (a)–(c) of Fig. 5, with panels (d)–(f) giving comparable uniform growth rates. The first thing to notice is that, locally, the domains in (a)–(c) are all growing linearly within the region where only a single spike is stable, independently of the speed of the growth. Such a linear growth is an emergent characteristic of the choice of *S*(*u*, *v*), with *S* not explicitly depending on time. Overall, the profiles of the cases of uniform exponential growth and concentration-dependent growth are remarkably similar. We also see that the spikes in (b) and (c) are at a much higher amplitude, and are much more localized than in the other simulations. This effect is due to local contraction, which is, in some sense, the opposite of the dilution effect observed in Fig. 2(f) and leads to larger amplitudes over smaller regions.

**Fig. 6:**
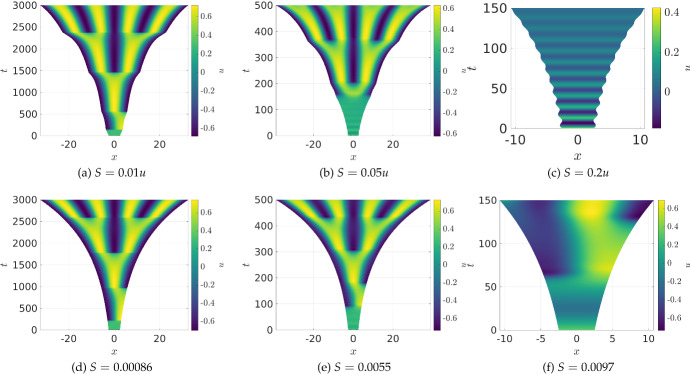
(Color Figure Online) Values of *u* from 1D simulations of the FitzHugh–Nagumo kinetics (25) under different growth scenarios. In all simulations the initial domain length is $L=5$, with $a = 1.01$, $b = 1$, $c = 1$, $i_0 = 1$, $D_1=1$ and $D_2=2.5$. The timescale and growth rates in (a)-(c) are chosen to match those in (d)-(f), so that the final simulation time is on a domain of exactly the same size

Lastly, we consider the FitzHugh–Nagumo kinetics (25) in order to explore the interplay of homogeneous oscillations and pattern formation in the concentration-dependent setting. We give three examples of simple linear functions *S*(*u*) in Fig. 6, though note that since *u* represents a voltage, it can be both positive and negative, and so this results in local domain growth and contraction. For slow growth, panels (a) and (d) show qualitatively similar behaviour, though note that (a) has changes in growth rate as the number or position of spikes change as in Fig. 5(a)-(c). For larger growth rates, however, we observe that the concentration-dependent case in (b) undergoes a period of rapid homogeneous oscillations before undergoing pattern formation, whereas the comparable uniform simulation in (e) has a shorter period of longer oscillations before forming patterns. Finally, in (c) we see that for sufficiently fast growth, rapid homogeneous decaying oscillations lead to periods of growth and contraction (with an overall growing tendency), whereas in (f) we observe one period of oscillation before the beginning of patterning takes places.

The interactions between homogeneous oscillations, particularly those coming from a Hopf bifurcation of the kinetics (25) are well-studied, e.g. see (Krause et al. 2019) and the references therein. The important contrast between the uniform growth and concentration-dependent cases can be in part explained by considering the base state given by (17). In particular, for all parameters in Fig. 6, there is a stable spiral steady state of the kinetics located at $(u^*,v^*)=(0,v^*)$ for some *S*-dependent $v^*>0$. However for the linear choice of *S* in panels (a)-(c), this stable spiral has a much larger imaginary eigenvalue, corresponding to higher frequency temporal oscillations, as observed. In fact the concentration-dependent case also undergoes a Hopf bifurcation case for slightly larger growth rates, leading to purely periodic growth and contraction as in the early time shown in (c). Using the inequality in Theorem 1, we find that panel (a) and the uniform growth cases in panels (d)-(f) all exhibit a growing range of unstable wavemodes with $k=0$ stable, whereas panels (b) and (c) exhibit an increasing range of unstable wavemodes including $k=0$ so that the base state is itself unstable, and whether or not a spatial pattern emerges is due to nonlinear competition between modes. While the precise condition determining whether or not an inhomogeneous perturbation leads to sustained amplitude patterns is not precisely determined by the Conditions of Theorem 1 (this is determined by nonlinear mode competition), one can gain insight into the impact of growth on the dynamics of the spatially homogeneous state given by (17).

## Two-Dimensional Simulations

**Fig. 7:**
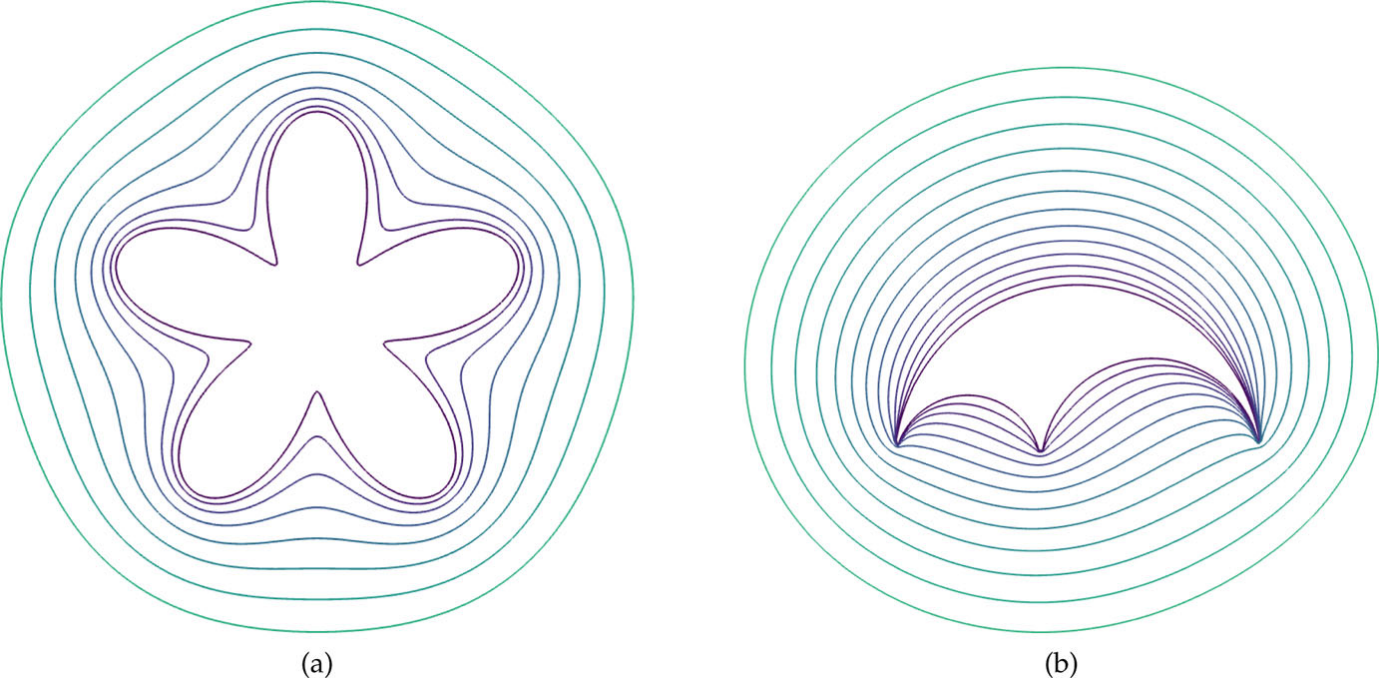
(Color Figure Online) Two examples of the growth of a boundary with the locally uniform expansion rate $S = 0.001$. In (a) we start with a starfish-like domain given by equation (13) in Krause et al. (2021) for $\gamma =0.8$ and $L=1$. In (b) we start with an Arbelos-like domain composed of the boundaries of three semicircles of radius 1, 2/5, and 3/5, respectively, with some truncation done near the lower boundary to prevent issues with extremely small finite elements. Boundary curves shown are arranged so that larger enclosed areas correspond to later times, with times uniformly sampled

Next we consider 2D simulations of concentration-dependent growth using the formulation given in Sect. 2.2. We implemented Eqs. (1) and (9)-(15) in the finite-element software COMSOL. The domain was discretized using second-order triangular finite elements, with timestepping done via a generalized backwards-difference formula of orders 1 to 5 with a tolerance set at $10^{-4}$. Initial steps within each iteration were taken as $10^{-8}$, with a maximum step constraint taken as 1 or 0.1 to confirm accurate resolution of the domain growth. As in the 1D case, each simulation was broken into a number of shorter iterations using automated remeshing via MATLAB LiveLink, with concentrations interpolated onto a new Lagrangian domain at the end of each iteration. In the 2D examples, we fixed the mesh size parameters (maximal and minimal element sizes, growth rates, etc.) so that domain growth tended to increase the number of elements used. Checks were carried out in the mesh size parameters, number of iterations used, and maximum timesteps taken to ensure convergence for specific simulations. All code and associated documentation can be found at Krause et al. (2022).

We begin by showing how the constitutive assumptions on the flow impact uniform growth rates where *S* is constant. Importantly, unlike in past work on uniformly growing domains (Plaza et al. 2004; Krause et al. 2019; Van Gorder et al. 2021), a locally uniform expansion of the domain does not lead to a uniform dilation given our assumptions on the flow $\varvec{a}$ corresponding to normal growth at the boundaries. We give two examples of this in Fig. 7, where larger enclosed areas correspond to the domain at later times. Here, we see that two highly non-convex domains both grow in a manner where curvature is uniformized at the boundary. This is remarkably similar to curve-shortening flows, which are well-studied in geometric analysis (Chou and Zhu 2001) and are related to other kinds of manifold evolution, such as the famous Ricci flow (Chow et al. 2006). It is also this quality, that uniform local growth does not lead to isotropic dilation, that prevents the use of the linear stability analysis given in Sect. 3 beyond 1D.

### Stable Inhomogeneous Solutions in Scalar Bistable Equations

**Fig. 8:**
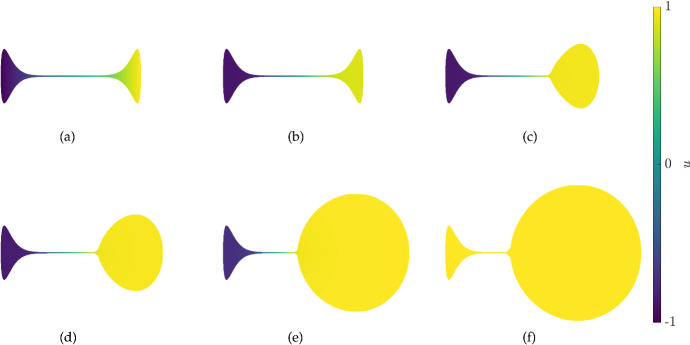
(Color Figure Online) Values of *u* from 2D simulations of the scalar bistable kinetics (27) in the dumbbell-shaped domain given by (29) with the diffusion parameter $D=1$ and growth rate $S = 0.000125(1+\tanh (50(u-0.9))$. Iterations are shown at times $t=0, 8, 2392, 3192, 4792,$ and 5272

**Fig. 9:**
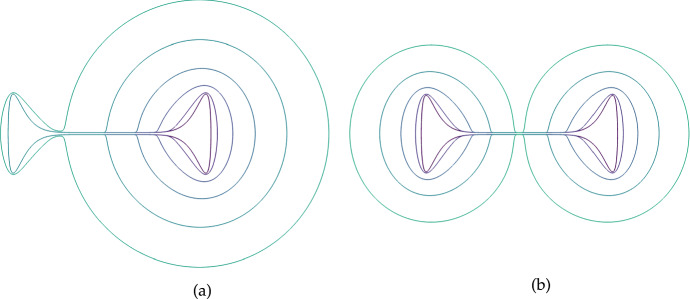
(Color Figure Online) Plots of the domain boundary from 2D simulations of the scalar bistable kinetics (27) in the dumbbell-shaped domain given by (29) with the diffusion parameter $D=1$. Panel (a) corresponds to Fig. 8 with $S = 0.000125(1+\tanh (50(u-0.9))$, and panel (b) to a simulation with $S = 0.000125(1+\tanh (50(|u|-0.9))$. Boundary curves shown are arranged so that larger enclosed areas correspond to later times, with times uniformly sampled

We next consider a simple example of concentration-dependent growth for a scalar reaction–diffusion equation, namely using the bistable kinetics (27). It is known that scalar reaction–diffusion equations with Neumann data cannot admit stable inhomogeneous steady states on 1D or general convex domains (Casten and Holland 1978; Matano 1979) and in fact this restriction of long-time behaviour even generalizes to time-dependent semilinear reaction–advection–diffusion problems (Hess 1989). In contrast, for non-convex domains bistable (or more generally multistable) kinetics can be used to make ‘locally’ stable regions separated by thin channels which are inhomogeneous, and there is a range of literature exploring these kinds of structures in many settings, for example (Matano 1979; Matano and Mimura 1983; Ward and Stafford 1999). We explore a few simple scenarios of concentration-dependent domain evolution to see what happens to such heterogeneous fronts.

We parameterize an initial dumbbell-like domain as,

29 $$\begin{aligned} x(s) = (s^8+0.01)\sqrt{1-s^8}, \quad y(s) = -(s^8+0.01)\sqrt{1-s^8}, \quad s \in [-1,1], \end{aligned}$$

and take as an initial condition $u(0,x,y) = x$, so that the concentrations approximately equilibrate to the value of $u= -1$ in the left region and $u=1$ in the right, with some diffuse boundary in the thin channel between them and parts of lobular regions near this channel. See Fig. 8(a) for this initial condition, which only changes slightly from the quasi-static value 8 time units later in panel (b). In subsequent panels of Fig. 8, we show domain growth that occurs where *u* is approximately greater than 0.9, and hence is localized in the rightmost region of the domain. Eventually this region grows so large that the small boundary to diffusion imposed by the thin channel is insufficient to prevent this steady state from overcoming the $u=-1$ state, and the entire domain approaches $u=1$, as would happen on a convex domain. Given the slow-growth timescale, we anticipate this destabilization of the inhomogeneous steady state occurs when the geometry destabilizes the heterogeneous front. This boundary could in principle be computed (Gokieli and Varchon 2005), but we do not do so here.

We also consider a growth function *S* where the domain grows for $|u|>0.9$, and compare this to the simulation in Fig. 8 by plotting the boundary of these two cases in Fig. 9. In panel (a), *S* is positive during most of the simulation time only in the rightmost region when *u* is near the value of 1. After the whole domain reaches this value, as shown in Fig. 8(f), both regions and the (now quite small) intermediate channel both grow. In contrast, the simulation in panel (b) of Fig. 9 never reaches a uniform value of *u*, and both regions expand uniformly as shown but maintain the values of $u\approx -1$ on the left and $u \approx 1$ on the right.

Intriguingly, if the growth is reversed so that the domain locally shrinks when $u>0.9$ (that is, we set $S \rightarrow -S$ from the simulation in Fig. 8), the rightmost region shrinks slightly, but it eventually reaches a state where $0< u <0.9$ and the growth appears to halt asymptotically, due to diffusion across the thin channel reducing the value of *u* in the rightmost region. At $t=120,000$, the maximal value of *u* is approximately 0.836 and the overall contraction rate of the domain is substantially smaller – the ratio of the integral of *S* at $t=8$ divided by the integral of *S* at $t=120,000$ is approximately 353, indicating substantially slower contraction at the later time.

### Concentration-Dependent Growth in 2D Gierer–Meinhardt Systems

**Fig. 10:**
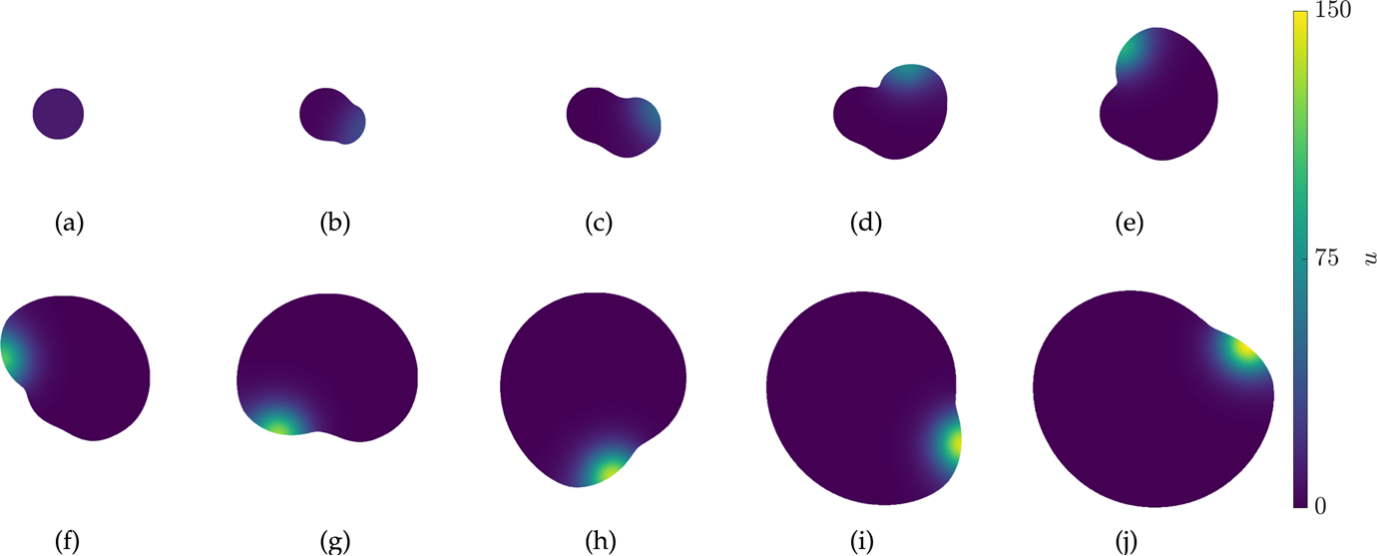
(Color Figure Online) Values of *u* from 2D simulations of the Gierer–Meinhardt kinetics (24) in an initially circular domain of radius 3. The parameters used are $D_1=1$, $D_2=1000$, $a=0.01$, $b=0.5$, $c=5.5$, with a growth rate of $S = 0.001(1+\tanh (100(u-22))$. Iterations are shown at equally spaced times with all panels using the same spatial scale

**Fig. 11:**
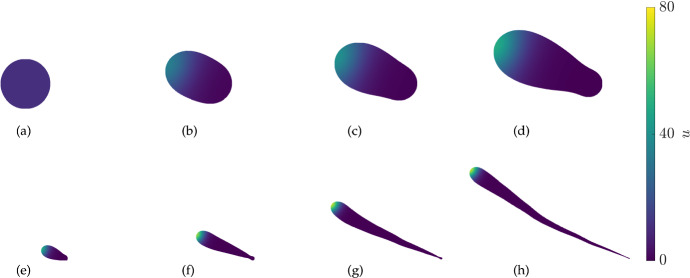
(Color Figure Online) Values of *u* from 2D simulations of the Gierer–Meinhardt kinetics (24) in an initially circular domain of radius 3. The parameters used are $D_1=1$, $D_2=1000$, $a=0.01$, $b=0.5$, $c=5.5$, with a growth rate of $S = 0.001((u/u^*)^2-1) = 0.001((u/11.02)^2-1)$. Iterations are shown at equally spaced times, with panels (d) and (e) being the same plot but resized so that panels (a)-(d) are shown on the same scale and panels (e)-(h) are shown on the same scale

**Fig. 12:**
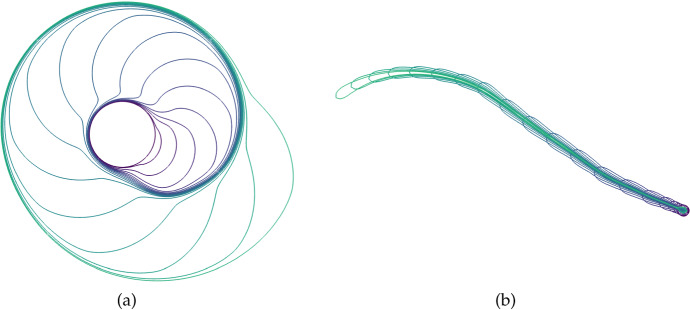
(Color Figure Online) Plots of the domain boundary from 2D simulations of the Gierer–Meinhardt kinetics (24). Panel (a) corresponds to Fig. 10 and panel (b) to Fig. 11. Boundary curves shown are arranged so that larger enclosed areas correspond to later times, with times uniformly sampled. In panel (b), later boundary curves are to the left but do not enclose larger areas due to contraction towards the right side of the domain

Next we consider an example of growth in the Gierer–Meinhardt system given by (1) and the kinetics (24). We choose parameters so that growth is localized at a peak, and a sufficiently large diffusion ratio so that there is only one low-amplitude peak for a fixed circular domain. We use initial conditions as in the 1D setting of the form $u(0,x,y) = u^*(1+\eta (x,y)), v(0,x,y) = v^*(1+\xi (x,y))$ where for each spatial point (*x*, *y*), the random variables $\eta$ and $\xi$ are normally distributed with zero mean and variance $10^{-2}$.

We show the evolution of the domain and concentration of *u* in Fig. 10. From panels (a) and (b) we can see that there is initially growth at a particular point on the boundary determined by where the localized spot initially forms. From the remaining panels we can see that this spot curves around the domain, presumably following regions of high local curvature as has been shown in Gierer–Meinhardt systems on fixed domains (Iron and Ward 2000). The interplay between local growth and curvature-based movement leads to a spiralling motion of the spike around the original circular domain. We show the boundary curves corresponding to this case in Fig. 12(a), where we see a strikingly seashell-like pattern of growth.

Next we consider a case of domain growth and contraction. As before we consider growth near the peak of the activator *u*, but assume the domain contracts away from this peak. We show plots from this case in Fig. 11. The short time dynamics are similar to the previous example, with a spot forming in one part of the domain and leading to a local protrusion between panels (a) and (b). However panels (c) and (d) clearly show that the domain is contracting away from the localized spot, leading to a fairly irregular shape. Over longer timescales the spot continues to grow whereas the ‘tail’ region left behind shrinks leading to the strange shape given in panel (h). Over longer timescales a spot eventually forms in the bottom-right corner of panel (h), which then also begins growing. We show a plot of the boundary curves in this case in Fig. 12(b), but note that unlike all previous boundary curves in this paper, these involve intersections rather than monotonically growing regions. These intersections correspond to contraction of the domain with boundaries from earlier times.

## Discussion

Primarily motivated by realistic coupling of domain evolution and morphogen dynamics in reaction–diffusion models of pattern formation, we have presented and explored a class of such models on domains generated by concentration-dependent growth. As one might expect, the possible dynamics in these cases can involve rich interactions between the domain and the reaction–diffusion system, leading to intricate bifurcation structures and captivating imagery, such as in the spiral of Fig. 12(a). Here we overview some of the key insights of this study, and outline potential fruitful directions for further work.

Considering domain evolution as a local process which expands or contracts space as defined by (3)-(4), there is essentially a unique 1D formulation of the model which we gave in Sect. 2.1, and which was previously studied by Seirin Lee et al. (2011b). Here we considered this framework in regimes of large growth, and also in regimes of growth and contraction, highlighting complexity that can emerge due to the interactions of both domain size and local dilution. In particular, we demonstrated that while robust spike-doubling still typically occurs for sufficiently slow growth, fast concentration-dependent evolution, or domain contraction, could lead to a variety of unexpected phenomena. These include: modification of travelling-wave dynamics as in Fig. 1; saturating plateaus of activator as in Fig. 2(f); bifurcations between growing, oscillating, and shrinking domains as in Fig. 4; apparent locally-in-time linear growth as in Fig. 5; and additional complexity of the background state, given in Equation (17), as in Fig. 6. We also performed linear stability analysis of this 1D model, emphasizing that it can be useful as a heuristic but provides minimal insight in complex growth regimes. Importantly, unlike in work by Van Gorder et al. (2021) and others, there is no obvious extension of these linear stability results to higher-dimensional growth for these kinds of local-growth models due to the nontrivial choice of constitutive assumptions for $N>1$.

In two and more dimensions, specifying the local dynamics of the domain is insufficient to prescribe the domain’s evolution in time. Here, we have posed irrotational growth and no tangential movement along the boundary, which may be interpreted as the flow being generated *solely* by point sources of density scaling with *S* and the impact of the boundary constraint (Batchelor 1967). We demonstrated that this leads to domains that reduce boundary curvature over time for constant uniform local-growth rates in Fig. 7. In this 2D setting, we also demonstrated how even simple growth dynamics can give rise to nontrivial domain restructuring in Fig. 10, and that growth and contraction can lead to more exotic phenomena as in Fig. 11.

We anticipate that many more interesting phenomena can be found, or constructed as in Woolley et al. (2021), and have made available an open source MATLAB code that can rapidly simulate these systems in 1D (Krause et al. 2022), as well as a COMSOL LiveLink code in the 2D case. In the 1D setting especially, we anticipate that the autonomous system given by (6)-(7) can be investigated directly using various tools from nonlinear dynamics and the analysis of PDEs. If *S* does not depend explicitly on time, as in all of the simulations reported here, the system is autonomous and in principle not much more complicated than many existing models of nonlinear diffusion and non-diffusible morphogen systems. In principle tools such as shadow-limits, spatial dynamics, and numerical continuation can all be applied to such a system. The travelling-wave case studied briefly in Sect. 4.1 is one topic of current further work, and highlights several nontrivial aspects of this autonomous yet complicated concentration-dependence. In addition to past work on spike-splitting events, mesa-splitting patterns like those seen in Figs. 3(h)-(i) and 6, have also been studied analytically in the slow-growth regime (see Figure 1 and the analysis in Kolokolnikov et al. (2007)).

While the 1D setting is interesting and plausibly tractable to various kinds of mathematical analyses, we also highlighted stark differences between both the modelling and such analysis in 1D and models with higher spatial dimensions. These raise important questions regarding appropriate tissue-mechanical constitutive assumptions, which can have nontrivial impacts on the dynamics in 2D and 3D models, and where any mathematical analysis becomes substantially more complicated even if mechanical deformations are essentially neglected, as in our simple constitutive model of the flow $\varvec{a}$. Related to this, many of the important insights regarding growth, such as the celebrated spike-doubling robustness shown by Crampin et al. (1999), hold true in 1D models but may not lead to robust patterning in higher-dimensional settings even in slow-growth regimes. For example, 2D stripes can undergo breakup or zigzag instabilities, plausibly ruining simple predictions of pattern-doubling (Kolokolnikov et al. 2006; Krause et al. 2019). More work needs to be done in understanding which insights from simple 1D models may be applicable to realistic tissue geometries, especially for scenarios where morphogens influence the geometrical evolution of the domain.

## Footnotes

1 The governing equations can be posed for general *N*-dimensional manifolds, though we will only pursue numerical simulations for flat one- and two-dimensional domains.

2 The last equality here is only unique up to a sign—the choice of the positive sign preserves orientation between the Lagrangian and Eulerian frames.

3 In fact, one can derive the base state system (17) by assuming that we are interested in perturbations of this form and collecting the *O*(1) terms.
